# What About the Girls? Exploring the Gender Data Gap in Talent Development

**DOI:** 10.3389/fspor.2019.00003

**Published:** 2019-07-11

**Authors:** Orlaith Curran, Aine MacNamara, David Passmore

**Affiliations:** ^1^School of Health and Human Performance, Dublin City University, Dublin, Ireland; ^2^Institute for Sport and Health, University College Dublin, Dublin, Ireland; ^3^Institute of Coaching and Performance, University of Central Lancashire, Preston, United Kingdom

**Keywords:** female athlete, youth athlete, gender gap, evidence-base, talent

## Abstract

Although there is an extensive literature about talent development, the lack of data pertaining to females is problematic. Indeed, the gender data gap can be seen in practically all domains including sport and exercise medicine. Evidence-based practice is the systematic reviewing of the best evidence in order to make informed choices about practice. Unfortunately, it may be that the data collected in sport is typically about male experiences, and not female; a rather unfortunate omission given that approximately half of the population is made up of women. When female athletes are underrepresented in research there are issues when making inferences about data collected in male dominated research domains to inform practice and policy for female athletes. In parallel, female sport participation is continually increasing worldwide. Recognizing the importance of evidence-based practice in driving policy and practice, and reflecting the gender data gap that is a consistent feature of (almost) all other domains, we were interested in examining whether a gender data gap exists in talent development research. The results suggest that a gender data gap exists in talent development research across all topics. Youth athlete development pathways may be failing to recognize the development requirements of females, particularly where female sports may be borrowing systems that are perceived to work for their male counterparts. In order to ensure robust evidence based practice in female youth sport there is a need to increase the visibility of female athletes in talent development literature.

## Introduction

The literature base, and data, about talent development in sport is extensive and includes a range of empirical articles (Coutinho et al., 2015; Forsman et al., 2016), theory-driven papers (Phillips et al., 2010; Davids et al., 2013), and models of talent development (Gagné, 2004; Bailey and Morley, 2006) that are purported to enable researchers, practitioners, and policy makers generate a clear understanding of what is known in order to guide their practice and inform policy decisions. Indeed, across all sport science disciplines there is an understanding of the importance of evidence-based practice in determining the best outcomes for athletes and coaches. Evidence-based practice is the systematic reviewing of the best evidence in order to make informed choices about practice. Unfortunately, it may be (as elsewhere in a data-driven world) that the data collected in sport is typically about male experiences, and not female; a rather unfortunate omission given that approximately half of the population is made up of women (The World Bank, 2017)! The gender data gap (Perez, 2019) is important to consider against the growth of womens' and girls' sport in general and the subsequent implementation of female specific talent development pathways (e.g., The Football Association Girls' England Talent Pathway; The FA, 2017). If data is used, in the talent development space, to drive decisions about resource allocation, pathway structures, coaching, and competition about female sport, are we sure that it reflects the needs of specific populations?

There has been much recent attention on the gender data gap across domains from medicine (Vitale et al., 2017), to vehicle safety (Linder et al., 2011), and urban design (Carpio-Pinedo et al., 2019). Simply, researchers fail to collect data on women yet the results of research are extrapolated to females without due consideration of the impact of that transfer (Perez, 2019). When women are underrepresented in the data that underpins how decisions are made, when the statistics ignore them, the results can be problematic. For example, women are more likely to be misdiagnosed of a heart attack as they experience different symptoms to men. However, cardiac trials generally use male participants leading to the most common known symptoms of cardiac events to be those experienced by males. Similarly, cars are designed around the body and physical profile of a male, increasing the likelihood of injury to women in collisions. The dangers of not having, and using, robust data on females is far-reaching.

Even when data on females is collected it is not always analyzed appropriately. As such, it is important to collect sex segregated data as “*women are not just men with boobs and tubes, they have their own anatomy and physiology that deserve to be studied with the same intensity*” (Mcgregor, 2015). Of course, in medicine and transportation safety the results of the data gap can be deadly (Bose et al., 2011; Linder et al., 2011; Vitale et al., 2017). In talent development, while there may not be the same catastrophic repercussions to excluding data on female experiences, failing to account for the experiences of females can result in inefficient talent systems and less than optimal experiences for female athletes. As such, closing the gender data gap requires the need to count female experiences explicitly in all fields. Sport and exercise medicine research has already been reported to significantly under-represent females in current literature, accounting for <40% of the total number of participants (Costello et al., 2014).

The last 20 years has seen large and robust literature emerge exploring various aspects of talent development (Coutinho et al., 2016; Rongen et al., 2018; Bennett et al., 2019). This research base has focused on a broad range of factors including identifying key aspects of the talent development environment (Wang et al., 2016; Li et al., 2017; Gledhill and Harwood, 2019), the importance of psycho-behavioral factors (Höner and Feichtinger, 2016; Erikstad et al., 2018a,b; Tedesqui and Young, 2018), physiological (Arazi et al., 2013; Forsman et al., 2016; Fornasiero et al., 2018; Jones et al., 2018), coaching (Romann et al., 2017; Peña-González et al., 2018), family (Domingues and Gonçalves, 2013; Elliott et al., 2018), and early experiences (Ford et al., 2009; Schorer et al., 2010; Coutinho et al., 2015) on the trajectory of young athletes. However, if the research-practice divide is to be effectively bridged for *all* athletes and robust implications for practice offered to practitioners, it is important to critically examine this evidence base and its applicability for both male and female athletes. Published literature such as journal articles present the knowledge base of a given discipline and reflect the discipline's history, trends, and research norms. As such, before research findings can be applied with confidence to particular contexts, it is important to establish that the research reflects *that* context.

Funding and structures for women's sport is increasing across the world with the establishment of professional leagues in, for example, soccer, hockey, athletics, and talent development pathways and academy structure for young female athletes developing in parallel. Often, the structures designed for female athletes have been “borrowed” from their male counterparts, perhaps without due interrogation of the similarities and differences that may exist between the two cohorts. Recognizing the importance of evidence-based practice in driving policy and practice, and reflecting the gender data gap that is a consistent feature of (almost) all other domains, we were interested in examining whether a gender data gap exists in talent development research. Therefore, the purpose of this paper was 2-fold. Firstly, the aim of this study was to review the peer-reviewed literature in talent development published between 1999 and 2019 to examine whether female athletes were represented in the participant sampling. Building on this, the second part of the paper discusses some reasons why future studies with female athletes are important in order to ensure that the literature is reflective of commonalities and differences in their experiences.

## Methods

### Development of Search Strategy

As scientist-practitioners our aim is to generate practically meaningful knowledge. As such, this study was underpinned by a pragmatic research philosophy (Giacobbi et al., 2005) and this philosophy guided all parts of the research process. This literature search utilized in this study employed review principles similar to conventional systematic reviews in order to ensure that adequate selection of literature based on replicable criteria occurred (Smith, 2010). A list of key words relevant to the aim and theme of the research was created (Smith, 2010) and these search parameters were trialed in a preliminary search on the SPORTDiscus database. During this preliminary search, every 10th result was checked and analyzed for relevance and to consider whether additional keywords should be included. This process was repeated until the most effective search terms were identified (i.e., the terms that returned the most relevant and specific literature in relation to the research question). Irrelevant terms that repeatedly came up in the search results were excluded (i.e., injury). Following this process, the final list of search terms included the following:

**Table:** 

“Talent Development” OR “Talent Identification” OR “Talent Selection” OR “Talent” OR “Long Term Development” OR “Specialisation” OR “Relative Age Effect” AND “Youth Sport” OR “Youth Athlete” OR “Young Athlete” OR “Adolescence” AND “Maturation” OR “Growth” AND “Psychology” OR “Mental Skills” NOT “Injury”

In the final literature search two relevant databases, SPORTDiscus and Ovid, were broadly, though not exhaustively, searched using the key words in different combinations to allow for the return of relevant research papers.

### Inclusion/Exclusion Criteria

Inclusion and exclusion criteria were employed to create clearly defined boundaries for the literature search (Smith, 2010). The inclusion criteria were, (a) peer reviewed empirical research studies, (b) published from January 1999 until April 2019 (when the formal search was finalized), (c) in English language, (d) have gathered original qualitative or quantitative evidence from young athletes only (under 21 years of age), and not evidence from other stakeholders (e.g., coaches, parents, peers etc.), that facilitate talent development knowledge and understanding, (e) provide information on the age and gender of the research participants, and (f) contain specific reference to either talent/talent development/talent identification/talent specialization, long term development/growth/maturation, or psychological skills/psychological attributes to talent development within the title, abstract or listed key words.

### Search Returns

**Figure 1:**
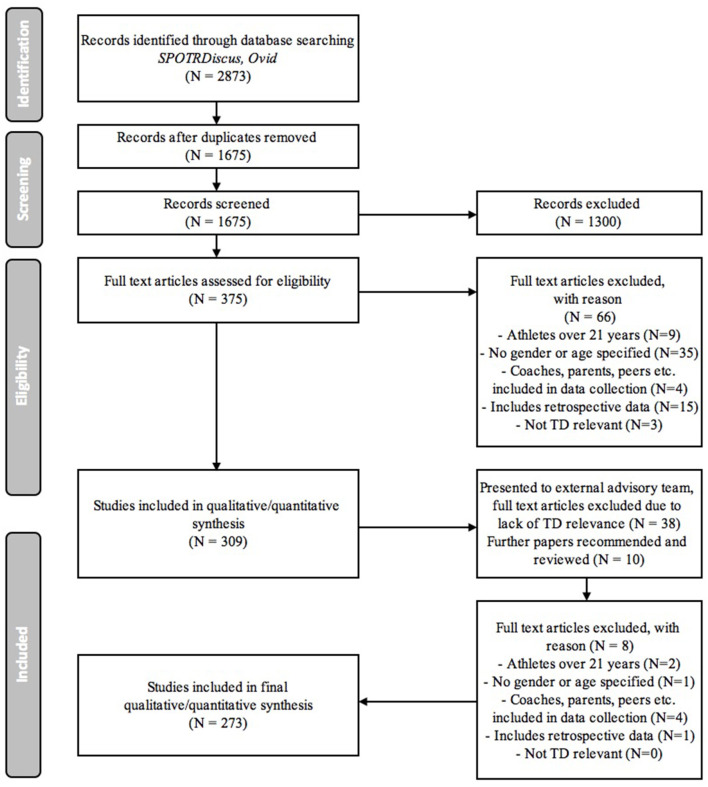
PRISMA flow diagram of study selection.

The search process came to a close on the 1st of April 2019 and retrieved 2,873 potentially relevant hits. Duplications were removed and abstracts and titles assessed for relevance. Based on the inclusion/exclusion criteria, 2,498 search returns were excluded and 375 papers kept for full-text retrieval. Most studies were excluded due to duplicates, a lack of definitive relevance to talent development, or their focus on senior (above 21 years of age) elite athletes. After full-text retrieval and review, 312 of the 375 papers met the inclusion criteria. Most studies were excluded due to the inclusion of coaches or parents in the participant group, no clearly defined age or gender of participants as well as inclusion of participants above 21 years. This reference list was examined by an experienced external advisory team. Suggestions from this advisory team included the removal of further studies due to a lack of definitive talent development focus as well as the suggestion for consideration of additional references. These additional studies were considered and 10 papers accessed and reviewed. Following this process an additional two references were added. Following this process, 276 studies met the inclusion criteria and were analyzed for the purpose of this review. Following the PRISMA flow diagram guidelines developed by Moher et al. (2009), an outline of the detailed overview of the search process, along with the reasons why papers were rejected, can be found in Figure 1.

### Data Synthesis

The literature search was used to identify and elicit research papers regarding areas within talent development of athletes under 21 years of age. The aim of the literature search was to examine whether a gender data gap was apparent in talent development research carried out from 1999 to 2019. As a first step, the first author went through an extensive process to check all papers for relevance and identify any alternative and appropriate key words for use within the literature search to ensure accuracy and comprehensiveness. A content analysis was used to extract key information from the data regarding the gender, age and the key words used within each research paper (Pope et al., 2007).

### Establishing Trustworthiness

To establish trustworthiness and meet the criteria of validity and credibility, a number of processes were followed (Creswell and Miller, 2000; Sparkes and Smith, 2009). Firstly, *peer debrief*, which involved a consistent review of the research process by an experienced supervisor who offered their support and criticisms, was employed (Creswell and Miller, 2000). Peer debrief took place regularly (i.e., every 2–4 weeks) through meetings and informal discussions. Secondly, an *advisory team*, comprised of two external researchers who had previously published studies within the explored literature, was established (Smith, 2010). The advisory team was provided with references of included studies, strategies for developing the research question, inclusion and exclusion criteria, and a briefing about the purpose of the literature search. The included papers and research methods employed were approved by the panel and suggestions for additional inclusions provided.

## Results

Reflecting the aims of this study, we were particularly interested in examining the participant populations included in the talent development research from 1999 to 2019. Table 1 illustrates the gender breakdown across the selected publications. Of the 276 research papers included in the data synthesis, only 9.42% included a female only population in comparison to 60.14% in a male only population, and 30.43% with both males and females included in the participant group (gender aggregated). This finding clearly indicates that a gender data gap exists in the talent development research from 1999 to 2019. Table 1 also presents the extent to which the gender aggregated research papers report findings on males and females considered together as a single participant group or if the findings were compared between the genders. 77.38% of the 84 gender aggregated papers present results where males and females have been considered separately. Of these, 86.15% illustrate a difference in the results relating to females in comparison to males.

**Table 1:** The number and percentage of gender groups (male, female, and gender aggregated; both male and female); and the number and percentage of papers where comparisons are made between genders represented in the research papers included in the literature search data set.

**Gender**	**Count**	**%**	**Gender comparison (aggregated groups)**	**Count**	**%**	**Reported difference between genders**	**Count**	**%**
Male	166	60.14	Yes	65	77.38	Yes	56	86.15
Female	26	9.42	No	19	22.62	No	9	13.85
Both	84	30.43	Total	84	100.00	Total	65	100.00

Having established that a gender data gap was apparent, we then reviewed the results to consider whether the data gap was more or less apparent in the literature pertaining to specific topics in talent development (see Table 2). Research relating to the relative age effect and maturation of youth athletes was the most represented topic in the literature search (36.96%) and, perhaps surprisingly, research relating to sport specialization (3.26%) was the least evident. Reflecting the purpose of the paper, each topic was then analyzed to examine whether a gender data gap was apparent and this examination found that females were underrepresented across every topic. Females accounted for only 3–17% of the participant groups in the included literature compared to a 38–73% representation for male only groups. Gender aggregated data (male and female participants) was also higher than the female only across all but one topic of the literature search. Physical factors returned 8.82% of studies for gender aggregated groups compared to 17.65% for female only groups. The gender aggregated group also accounted for less studies across all topics compared to the male only group.

**Table 2:** The number and percentage of topics and number and percentage separated per participant group represented in the research papers included in the literature search data set.

	**All**		**Male**		**Female**		**Both (*****M & F*****)**	
**Discipline/Topic**	**Count**	**%**	**Count**	**%**	**Count**	**%**	**Count**	**%**
Talent development (Tactical/performance focus)	19	6.88	14	73.68	2	10.53	3	15.79
Talent development (External to sport/environmental focus)	21	7.61	8	38.10	3	14.29	10	47.62
Relative age effect/maturation	102	36.96	58	56.86	8	7.84	36	35.29
Talent identification/selection	60	21.74	42	70.00	6	10.00	12	20.00
Sport specialization	9	3.26	6	66.67	0	0.00	3	33.33
Physical	34	12.32	25	73.53	6	17.65	3	8.82
Psychological	31	11.23	13	41.94	1	3.23	17	54.84

Finally, we examined the data to identify trends within talent development research published from 1999 to 2019. Table 3 highlights the growth of this particular area of research in more recent years. 38.41% of the included literature were published from 2016 to 2019 compared to only 7.61% of the studies published between 2007 and 2009. Only 5.80% of the papers included in the literature search were published between 1999 and 2006. Table 3 also highlights the gender breakdown of the published papers by year and presents a clear underrepresentation of research papers with female only populations. Although attention on topics pertaining to talent development has increased year-on-year, the gender data gap has remained consistent.

**Table 3:** The number and percentage of publications by year and number and percentage separated per participant group represented in the research papers included in the literature search data set.

	**All**		**Male**		**Female**		**Both (*****M & F*****)**	
**Year of publication**	**Count**	**%**	**Count**	**%**	**Count**	**%**	**Count**	**%**
2016–2019	106	38.41	58	54.72	8	7.55	40	37.74
2013–2015	86	31.16	55	63.95	9	10.47	22	25.58
2010–2012	47	17.03	28	59.57	5	10.64	14	29.79
2007–2009	21	7.61	17	80.95	1	4.76	3	14.29
1999–2006	16	5.80	8	50.00	3	18.75	5	31.25

## Discussion

The gender data gap represents an unequal representation of females across numerous domains in a world driven by data (Perez, 2019). Although there has been considerable growth in research relating to talent development in recent years, females are underrepresented in this data. Given that research should underpin advancements in real world practice in sport and youth athlete development, this gender data gap may have significant implications. Gender differences play an important role in the development of young athletes and there are notable differences between males and females in this regard; for example, physical (Batterham and Birch, 1996; Cramer et al., 2002; Weber et al., 2006; Bradley et al., 2014; Clarke et al., 2014), and cognitive (Crocker and Graham, 1995; Phillipe and Seiler, 2005; Murcia et al., 2008) differences between males and females are well-documented. Table 2 illustrates that the gender data gap is apparent across these important constructs. This becomes problematic when applying current findings to female development pathways and practices since gender can potentially influence youth athlete development. For example, the aerobic fitness evolution of young females progresses at slower rates to their male counterparts (Fornasiero et al., 2018). Furthermore, youth female athletes perceive their environment as a more task oriented climate (Murcia et al., 2008) and have a more long-term development focus than males (Li et al., 2018). Similarly, relative age effects are less pronounced in female sports, potentially due to maturational differences between females and males (Romann et al., 2018). As such, *if* data is to be used to inform talent development practice, there is a need for caution when making inferences about female athletes from male dominated research studies. Though beyond the scope of this review, we additionally recognize the complexity of these issues and the need for research to evolve further to adequately and appropriately represent individuals of all gender identities, whether they identify as men, women, or other. However, in this context we have delimited the analysis to male/female to reflect the categorization of competitive sport.

This literature search highlighted an underrepresentation of female data across *all* topics of talent development research and questions the extent to which the research *as it stands* can be extrapolated with confidence to female talent pathways. For example, there is less evidence on females in research specific to talent identification, physical and psychological development. Despite the dearth of female based talent development literature, there is continued growth worldwide of female sport, with sports participation amongst girls and women at an all-time high, female athletes are participating in record numbers (Fink, 2015). Research pertaining to male athletes cannot be *assumed* to relate to female athletes and the implications of applying such research findings to female sport are vast, creating talent pathways likely to be unreflective of female athlete needs.

Based on the evidence presented here, it can be hypothesized that current talent development pathways and systems for female athletes have been designed and developed based on male data. As such, talent development systems and structures for female athletes appear to lack a robust evidence base and may instead be the product of experience, gut feel, and tradition largely adopted from male athlete experiences. Simply, there is clearly a need for evidence of the experiences, requirements, and reflections of female athletes on the talent development pathway across all topics of talent development—physical, psycho-behavioral, talent identification etc. and a greater visibility of female athletes in the literature. The lack of data for females in talent development undermines the ability to understand the experiences of women and girls in sport and the constraints and opportunities they experience. Furthermore, this paper presents clear evidence that data collection in talent development is distorted by gender biases and how this negatively impacts the ability to design appropriate policies, structures, and systems for female athletes. In addition to the gender data gap, we would also argue that having no data, or poor data on issues that affect female athletes in particular is a significant issue; some issues (e.g., maturation, puberty, pregnancy, menstruation) clearly impact female athletes differently than their male counterparts and gender data would help us understand this better. In sum, there is a clear need for unbiased data in order to design talent development policies and practices—the gender data gap means that we only have a partial snapshot of the experiences and requirements of females in this space. Rigorous gender data will also allow sports to make informed decisions for females in sport and track the efficacy of talent development interventions.

## Data Availability

The datasets generated for this study are available on request to the corresponding author.

## Author Contributions

OC, AM, and DP contributed to the design and implementation of the research and contributed to the final version of the manuscript. OC performed the data collection and analysis.

### Conflict of Interest Statement

The authors declare that the research was conducted in the absence of any commercial or financial relationships that could be construed as a potential conflict of interest.
